# Training the Trainers: Building Capacity for Early Detection of First‐Episode Psychosis in Primary Care

**DOI:** 10.1111/eip.70238

**Published:** 2026-07-26

**Authors:** Cristiano Noto, Francisco Heitor Rosa, Heber Odebrecht Vargas, Mauro Porcu, Regina Célia Bueno Rezende Machado, Sandra Odebrecht Vargas Nunes

**Affiliations:** ^1^ Early Psychosis Group (GAPi), Department of Psychiatry Universidade Federal de São Paulo São Paulo Brazil; ^2^ Health Sciences Post‐Graduation Programme, Health Sciences Centre State University of Londrina Londrina Paraná Brazil; ^3^ Department Clinical Medicine State University of Londrina, UEL Londrina Paraná Brazil; ^4^ Department of Psychiatry State University of Maringá Maringá Paraná Brazil; ^5^ Nursing Department State University of Londrina Londrina Paraná Brazil

**Keywords:** capacity building, community health network, early detection, first‐episode psychosis, health education, healthcare professional, training of trainers

## Abstract

**Background:**

Early detection of first‐episode psychosis (FEP) is critical for improving long‐term outcomes. Training healthcare professionals to identify and intervene at the earliest stages is a key strategy for reducing the duration of untreated psychosis (DUP).

**Objective:**

To describe a capacity‐building programme, based on a ‘Training of Trainers’ (ToT) model designed to empower healthcare professionals to become multipliers of knowledge on early FEP detection within their communities.

**Methods:**

A 72‐h blended learning course using problem‐based learning (PBL) was delivered to 47 healthcare professionals to build competencies in FEP identification and referral.

**Results:**

The 47 participants reported an increase in self‐perceived confidence in identifying FEP symptoms. Participant satisfaction was high, with an average score of 9.0 out of 10.

**Conclusion:**

The ToT model is a feasible and well‐received strategy for training professionals in early FEP detection. Empowering professionals as educators is a critical step to strengthen community mental health and improve access to treatment.

## 1 Introduction

First‐episode psychosis (FEP) marks the onset of a psychotic disorder that can become chronic and treatment‐resistant (Prakash et al. 2021), often causing cognitive decline over time (Bora and Murray 2014). Early detection and intervention are crucial, as reducing the duration of untreated psychosis (DUP) significantly improves a patient's prognosis and functional outcomes (Johannessen et al. 2001; Oliver et al. 2018).

The disorder typically emerges during adolescence and early adulthood, presenting a critical window when psychosocial and pharmacological treatments are most effective (Lundin et al. 2024). Despite the potential for effective treatment, functional recovery in areas like employment and relationships is a major challenge. Poor long‐term outcomes and social isolation are often driven by factors like cognitive deficits and the challenge of achieving a timely diagnosis (Hall et al. 2019).

The ‘Training of Trainers’ (ToT) model is a strategy that equips a select group of participants with the skills and knowledge to effectively train others. This cascading approach allows expertise to be scaled quickly and sustainably throughout an organisation or community. In the early psychosis context, the ToT model emerges as an approach to expand knowledge on FEP, preparing healthcare professionals to become multipliers within their communities. Educating these professionals improves the detection and management of mental health disorders, helping to reduce the existing treatment gap (Koly et al. 2021). Within the context of the Brazilian healthcare system, where the reach of primary care is extensive, training professionals to act as multipliers represents a strategy with high potential impact for public mental health.

This report describes a training programme based on the ToT model, developed to enhance the knowledge and ability of healthcare professionals to identify early signs of FEP and refer patients to treatment.

## 2 Methods

### 2.1 Study Design

This study describes a capacity‐building programme for healthcare professionals based on a ToT model. The objective was to develop healthcare professionals' competencies for the early detection of FEP and to enable them to disseminate this knowledge within their communities and clinical practices.

#### 2.1.1 Participant Recruitment and Eligibility

Participants were recruited through institutional channels of the State University of Londrina (UEL).

#### 2.1.2 Ethical Considerations

This programme was conducted as part of institutional educational and extension activities at the participating universities. Participation was voluntary and all participants provided written informed consent for the collection and use of satisfaction and feedback data for research purposes. Patient data used in clinical case illustrations were anonymised and fictionalised to protect confidentiality. The study protocol was reviewed and approved by the Research Ethics Committee of the State University of Londrina (CEP/UEL) 30628120.9.0000.5231. The programme was also preregistered in the Brazilian Registry of Clinical Trials (ReBec) under number RBR‐3yt263y, entitled ‘Digital health interventions based on an app for smartphones or e‐book for health promotion’.

### 2.2 The Training Programme and Pedagogical Approach

The programme consisted of a 72‐h course combining synchronous (live online classes) and asynchronous (self‐study materials) activities. The full curriculum structure, including learning objectives, teaching methods and workload distribution across the five modules, is presented in Table 1.

**TABLE 1:** Curriculum structure of the 72‐h Training of Trainers (ToT) programme for early detection of first‐episode psychosis in primary care, based on the e‐book Educar para Prevenir: Saúde Mental em Foco (Vargas 2024).

Module	e‐Book chapters (asynchronous)	Learning objectives	Synchronous activities and assessment	Hours
Module 1–Mental Health Foundations	Ch. 1—Epigenetics and adverse childhood experiences Ch. 2—Post‐traumatic stress disorder	Describe the stress‐vulnerability model underpinning mental health disorders Identify how early adversity and genetic vulnerability interact to increase psychosis risk Recognise PTSD presentations in primary care	Synchronous webinar: stress‐diathesis model and FEP risk Group discussion: clinical cases linking childhood adversity and first psychotic episode Formative: case‐based reflection questions	14 h (4 h sync 10 h async)
Module 2—Mood and Anxiety Disorders	Ch. 3—Anxiety disorders Ch. 4—Obsessive‐compulsive disorder Ch. 5—Depressive disorders • Ch. 6—Bipolar disorder	Recognise and differentiate anxiety, OCD, depressive and bipolar presentations Apply differential diagnosis skills to distinguish mood/anxiety disorders from early psychosis Identify suicidal risk across diagnostic categories	Synchronous case‐vignette workshop: differential diagnosis Structured discussion: when to suspect prodromal psychosis in a mood presentation Formative: diagnostic accuracy exercise	18 h (8 h sync 10 h async)
Module 3—Psychotic Disorders and Early Detection of FEP [Key Module]	Ch. 7—Schizophrenia spectrum: definitions, symptoms, and monitoring tools	Identify the five symptom domains of schizophrenia‐spectrum disorders (DSM‐5) Apply structured symptom‐monitoring instruments (SCID‐based screening) Recognise prodromal and early warning signs of FEP Initiate and document timely referral to early intervention services Describe the role of DUP and its impact on clinical outcomes	Synchronous lectures: FEP phenomenology and DUP Role‐play: structured screening interview Case pathway mapping: RAPS/SUS referral network Guest lecture: CAPS coordinator Summative: referral decision accuracy test	20 h (14 h sync 6 h async)
Module 4—Complex Presentations and Vulnerable Populations	Ch. 8—Eating disorders Ch. 9—Substance use disorders and addictive behaviours Ch. 10—Suicide in children, adolescents and young adults Ch. 11—Neurodevelopmental disorders	Recognise comorbid presentations that may complicate or mask FEP Assess suicidal risk in patients with psychotic symptoms Identify substance‐induced psychosis and distinguish from primary FEP Apply a biopsychosocial framework to complex, multi‐diagnostic cases	Synchronous case‐based learning (CBL) sessions: multi‐morbidity Structured discussion: substance use and FEP Formative: clinical vignettes with multi‐morbidity	12 h (6 h sync 6 h async)
Module 5—Self‐Monitoring, Digital Tools and the Trainer Role	Ch. 12—Self‐monitoring guide for mental health Ch. 13—Mental health assessment via digital/AI tools	Use validated self‐monitoring tools to support early symptom recognition Critically appraise digital and AI‐based screening tools for mental health Design and deliver a brief educational session on FEP for peers Identify strategies for sustaining community FEP awareness	Synchronous micro‐teaching: participants deliver a 10‐min FEP awareness session Peer feedback using structured rubric Summative: 10‐point satisfaction questionnaire (Likert scale) + open‐ended comments	8 h (8 h sync 0 h async)
Total	13 chapters—e‐book Educar para Prevenir (Vargas 2024)	5 modules covering the full spectrum of mental health disorders with emphasis on FEP early detection	Blended: synchronous (live online) + asynchronous (self‐directed e‐book study)	72 h (40 h sync 32 h async)

*Note:* Module 3 is highlighted as the central module for FEP early detection competencies.

Abbreviations: async = asynchronous (self‐directed e‐book study), CAPS = Centro de Atenção Psicossocial (Psychosocial Care Centre), CBL = case‐based learning, DUP = duration of untreated psychosis, FEP = first‐episode psychosis, RAPS = Rede de Atenção Psicossocial, SCID = Structured Clinical Interview for DSM‐5, SUS = Sistema Único de Saúde (Brazilian Unified Health System), sync = synchronous (live online sessions), ToT = training of trainers.

The course was structured as follows:

1. Participants first reviewed content from an eBook developed specifically for the course and studied real‐world clinical cases.
2. Live online classes were conducted to discuss FEP diagnostic features, deepen the analysis of clinical cases and present the available community health network for Intervention

### 2.3 Training Content and Tools

#### 2.3.1 FEP Assessment and Screening

To enhance early FEP detection, participants were trained to use a structured screening questionnaire adapted from First et al. (2017). The tool consists of ‘yes’ or ‘no’ questions:

*Have you ever felt like people were talking about you or watching you in a special way?*

*Have you ever believed that something on TV, radio, or in a song was meant especially for you?*

*Have you ever heard voices that others could not hear?*

#### 2.3.2 Referral to Therapeutic Interventions

**FIGURE 1:**
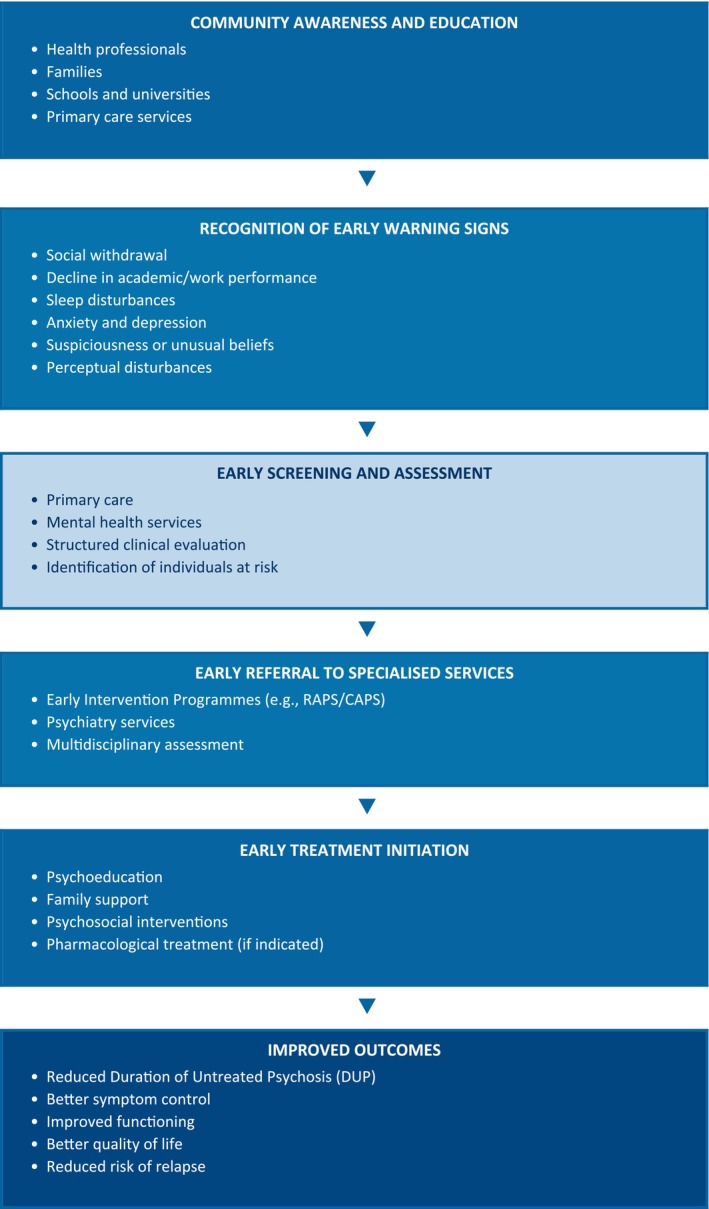
Early detection and intervention pathway for first‐episode psychosis. Early detection of first‐episode psychosis (FEP) involves sequential steps from community awareness and education to recognition of early warning signs, structured screening and assessment, prompt referral to specialised services and timely initiation of treatment. Successful implementation of each step can substantially reduce the duration of untreated psychosis (DUP) and improve clinical, functional and quality‐of‐life outcomes. CAPS = Centro de Atenção Psicossocial, RAPS = Rede de Atenção Psicossocial.

The training detailed the referral pathway to the Psychosocial Care Network (RAPS), which encompasses the sequential steps from community awareness to early treatment initiation (Figure 1) (BRASIL.Ministério da Saúde, Secretaria de Atenção à Saúde, Departamento de atenção Básica 2025). When FEP is suspected, patients are directed to services such as:

Psychosocial care centres (CAPS): specialised outpatient units for multidisciplinary follow‐up.

Therapeutic residential services: assisted‐living facilities that promote autonomy and social reintegration.

General Hospitals with Psychiatric Beds: for inpatient care during acute episodes.

### 2.4 Case‐Based Learning (CBL) Example

The following is an example of a clinical case discussed during the training to illustrate the practical application of concepts:

Problem: A 17‐year‐old male presented with a six‐month history of progressively worsening psychotic symptoms. His presentation was characterised by persecutory delusions and delusions of reference. The patient also experienced auditory hallucinations and exhibited disorganised behaviour, including an incident where he destroyed a television set, stating it was ‘talking about his life.’ This was accompanied by significant social withdrawal and a marked decline in functional abilities. Symptoms persisted despite the cessation of previous cannabis use, and his family history was positive for severe psychiatric illness.

Discussion: The case exemplifies a FEP consistent with a schizophrenia spectrum disorder, with symptomatology across multiple domains (delusions, hallucinations, disorganised thought, disorganised behaviour and negative symptoms). Cannabis‐induced psychosis was considered in the differential diagnosis but was deemed unlikely given the persistence of symptoms after substance cessation.

### 2.5 Programme Evaluation

Participant engagement, confidence and satisfaction were assessed using a course evaluation form developed specifically for this training and administered at the conclusion of the programme. No validated instruments, standardised questionnaires, objective performance measures, or formal competency assessments were employed. Participants provided self‐reported feedback regarding their perceived engagement with training activities, their confidence in recognising and addressing mental health concerns and their overall satisfaction with the course. Satisfaction was rated on a scale of 0 to 10. The data therefore reflect participants' subjective perceptions of their experience and do not permit conclusions regarding objective changes in knowledge or clinical competence.

## 3 Results

The ToT programme successfully trained 47 healthcare professionals from diverse fields, including medicine, nursing, psychology, social work, nutrition and speech therapy (comprising one nutritionist, one speech therapist and three social workers, with the remaining 42 participants drawn predominantly from medicine, nursing and psychology). The programme was delivered over a four‐month period through a blended format of asynchronous online materials and synchronous live sessions.

Participants subjectively reported high engagement throughout the course, based on qualitative self‐reported feedback collected via the course evaluation form. The use of real clinical cases as a pedagogical tool was particularly effective, allowing participants to directly apply theoretical knowledge to practical scenarios. At the conclusion of the programme, participants reported enhanced confidence and skills in several key areas addressed by the training:

Identifying and differentiating the subtle signs and symptoms of FEP.

Recognising critical factors for early detection, such as symptom duration and functional decline.

Understanding the role of family history and substance use in the diagnostic process.

Developing strategies for sensitive communication with patients and their families.

These areas represented the topics of greatest emphasis within the programme. Participants also reported perceived improvements across other mental health domains addressed in the training. As area‐specific numerical scores were not collected, a formal quantitative comparison of perceived improvement by domain cannot be provided; this is acknowledged as a methodological limitation.

The programme's success was further evidenced by a high level of participant satisfaction, which averaged 9.0 out of 10 in the final course assessment.

## 4 Discussion

This experience report demonstrates that a ToT model is a feasible and well‐received strategy for building capacity among healthcare professionals for the early detection of FEP. The programme's core strength lies in its dual objective: not only to equip professionals with clinical knowledge but also to empower them to act as educators within their communities, a critical step towards shortening the DUP (Johannessen et al. 2001). The high satisfaction scores suggest that the blended learning format and case‐based approach were effective in engaging participants and instilling confidence in their new skills.

While other capacity‐building initiatives have targeted early psychosis detection, our programme is unique in adapting the ToT model to the Brazilian context. This approach represents an innovative strategy to expand mental health literacy and strengthen referral pathways across primary care.

The implications of this training are significant, particularly because FEP typically emerges in late adolescence and early adulthood, a critical developmental period where early intervention can drastically improve long‐term outcomes (Lundin et al. 2024). Our model directly confronts the well‐documented treatment gap in mental healthcare. Globally, barriers to early treatment remain pervasive (Srihari et al. 2014). A ToT programme for healthcare professionals on early psychosis detection typically involves a multi‐faceted approach, focusing on enhancing diagnostic skills, improving referral processes to early intervention services and building capacity within the local healthcare system to deliver ongoing care and support (Lester et al. 2009).

Contrary to concerns sometimes raised in the literature, early detection and active case‐finding for FEP do not delay appropriate treatment‐seeking; rather, they facilitate timely access to care by reducing the period of unrecognised illness. Specifically, the World Health Organization, WHO (2022) reports that approximately one in eight people globally live with a mental disorder, yet a significant care gap persists, with only about 50% of affected individuals receiving treatment. Care seeking in individuals who experienced psychotic symptoms is often delayed due to unrecognised symptoms, confusion with mood disorders or typical adolescence and caregivers' limited support stemming from stigma and systemic barriers (Yarborough et al. 2019). This need is underscored by evidence that a psychotic disorder diagnosis was not given to 39% of subjects at their initial episode of care (Albin et al. 2021). Delayed treatment of psychosis, stemming from inadequate early intervention services, may contribute to a prolonged DUP (Malla 2022). Our training model directly addresses this gap by strengthening the capacity of the primary healthcare network to recognise and respond to early signs of psychosis.

### 4.1 Limitations

This report has several limitations. First, the study is explicitly framed as a feasibility and acceptability report. Engagement, confidence and satisfaction were assessed solely through a non‐validated, course‐specific evaluation form; no standardised instruments, objective performance measures, or formal competency assessments were employed. The data therefore reflect self‐reported perceptions rather than objective measures of learning or behaviour change. Confidence gains were not quantified using validated scales, and area‐specific numerical scores were not collected; consequently, a formal quantitative comparison of perceived improvement across individual domains cannot be provided. Furthermore, no pre‐course baseline measure was obtained, so changes in confidence cannot be attributed solely to the intervention, and the absence of pre/post knowledge assessments, validated psychometric instruments, or objective behavioural outcomes means that no causal claims regarding learning gains can be supported. Second, the downstream effectiveness of the trained professionals as disseminators of knowledge in their respective communities was not assessed and depends on external factors such as institutional support, community engagement and local resource availability. Third, the study did not measure whether the intervention ultimately led to a reduction in the DUP, which remains a critical outcome for future research. Fourth, the ToT and CBL pedagogical approaches require continuous training cycles and sustained institutional support to ensure that educators maintain the competencies necessary for effective knowledge dissemination; without such ongoing reinforcement, fidelity to the original training model cannot be guaranteed. Fifth, the real‐world impact of the programme ultimately depends on the sustained motivation of the trained healthcare professionals to actively transfer their acquired knowledge to community members and professional peers; this cannot be ensured by the training alone and is subject to individual and organisational factors beyond its scope. Sixth, identifying the most effective strategies for interaction and engagement between health professionals and community members in diverse primary care settings remains an ongoing methodological challenge that future implementation studies should address systematically. Seventh, the programme did not systematically record the numerical breakdown of participants by professional category; as a result, a detailed characterisation of the sample by discipline cannot be provided, which limits the interpretation of profession‐specific training needs.

## 5 Conclusion

This report underscores the value of the ToT model as a feasible and well‐accepted approach to training healthcare professionals in the early detection of FEP and empowering them to educate their communities. By creating a network of local champions, such programmes hold the potential to enhance mental health literacy, encourage help‐seeking behaviours and bridge the gap between symptom onset and treatment.

The ToT model, given its low resource requirements and adaptability to blended learning formats, may offer a viable framework for scale‐up within the Brazilian primary healthcare system. Replicability in other settings would require contextual adaptation and formal evaluation before broader claims can be made. Further research should build on these findings by implementing longitudinal studies to quantitatively assess the impact of this model on clinical outcomes, particularly whether this approach contributes to a measurable reduction in the DUP, which remains the ultimate benchmark of effectiveness.

## Funding

The authors have nothing to report.

## Conflicts of Interest

The authors declare no conflicts of interest.

## Data Availability

Data sharing not applicable to this article as no datasets were generated or analysed during the current study.
